# Editorial: Feast your eyes: diet and nutrition for optimal eye health

**DOI:** 10.3389/fnut.2025.1579901

**Published:** 2025-03-04

**Authors:** Pinakin Gunvant Davey, Arunkumar Ranganathan

**Affiliations:** ^1^College of Optometry, Western University of Health Sciences, Pomona, CA, United States; ^2^Department of Ophthalmology and Visual Science, John A. Moran Eye Center, University of Utah School of Medicine, Salt Lake City, UT, United States

**Keywords:** carotenoids, zeaxanthin, lutein, glaucoma, macular degeneration, cataract, myopia, diabetes

“Let food be thy medicine and medicine be thy food.” Hippocrates said these powerful words, and we have indeed strayed far from them in our world of convenience and fast-food diets. This Research Topic focused on arguably one of the most important senses—vision—and perhaps one of the most important topics—nutrition. How does one obtain and maintain optimal eye health? To answer this question, let us look at an example from the recent past. The Age-Related Eye Diseases trials 1 and 2 shed a lot of light on this topic, and have led to recommendations on carotenoid vitamin supplements for the prevention of age-related macular degeneration (AMD) (1–3). Various systematic reviews and meta-analyses have shown that, although effects may vary in different stages of AMD, carotenoid intake may benefit and improve vision in healthy eyes, and all stages of AMD stand to benefit from its intake (4–6). Its benefits are not limited to eyes but also organs like the brain (7–10) and heart (11) and disease states like diabetes (12, 13) that can lead to widespread inflammation. One simple takeaway can be that one needs to increase carotenoid intake. A wider perspective would be that “we are what we eat” and that many other aging and disease states can be controlled by diet.

To this accord, the studies in this Research Topic looked at a wide range of topics dealing with disease states that create burdens of colossal proportions like glaucoma, AMD, diabetic retinopathy, and cataracts. The studies do so by evaluating the effects of deficiencies in nutrients or analyzing a large sample cohort from the National Health and Nutrition Examination Survey (NHANES).

It is not an exaggeration to say we are having a myopia epidemic (14). Whereas in most countries emmetropia was normal, countries like China are now experiencing myopic refractive error becoming the norm (14). Obviously, genetics play an important role, but the effects of epigenetics cannot be underestimated, and researchers (Xiao et al.) have found, using the NHANES data, that cis-β carotene is significantly associated with the risk of myopia and high myopia. The results are indeed fascinating, as they did not find an association with other micronutrient vitamins such as A, D, E, C, α-carotene, trans-β-carotene, or lutein zeaxanthin. Another study performed by Xu et al., using the NHANES data 2005–2008 showed that, although vitamin D and magnesium were protective against diabetic retinopathy, the protective effect of vitamin D primarily benefited individuals deficient in magnesium. This highlights the complex interplay of nutrients in health and disease states. Bhandarkar et al. looked at low-carbohydrate, high-fat diets in individuals with diabetes and found health benefits going beyond glycemic control, including changing the lipid profile and ocular inflammatory biomarkers. Although not significantly correlated to Hb a1c, this study indeed sows seeds for the need for further investigation in this area, as various biomarkers, such as ICAM-1, IL-17A, IL-1β, and TNF-α, all showed clinically significant changes during the follow-up.

Cataracts is one of the reversible causes of vision loss (15). Most of us have accepted that a complex set of circumstances eventually causes a naturally occurring clear crystalline lens to opacify. If we are what we eat, can there be nutritional methods to prevent the formation of cataracts? Zhang et al., and Feng et al., add to the body of literature already present in this area. Zhang et al., summarized the current body of work in their mini review article, whereas Feng et al., found that famine exposure during the early stages of life is associated with a heightened risk of developing cataracts. This brings another question to mind: although we all would like to be citizens of planet earth, there are significant dietary constraints in various countries and one plan to fix the nutritional requirements of all countries would not work. Thus, studies such as those of Thirunavukkarasu et al., and Nuredin et al., highlight the need for targeted nutritional intervention measures to improve vision health in different regions.

Lastly, AMD (16) and glaucoma (17) are not only the leading causes of irreversible blindness but also pose a huge socioeconomic burden. The current therapy for glaucoma is to lower intraocular pressure (IOP); however, lowering of IOP does not guarantee halting disease progression. Further, glaucoma patients are on multiple medications and require surgery. Both AMD and glaucoma are at high proportions due to the aging population (16–18). Thus, it remains important to look for alternative and adjunct therapies for both these disease states. The work from Yang et al. on vitamin B6 points to a lower risk of glaucoma in the highest quartile and Lee et al. shows that higher intake of omega-3 long chain polyunsaturated fatty acids decreases the risk of AMD. These are easy for patients to implement and, furthermore, these nutrients have many other benefits to the body that also contribute to overall health.

To say that we live in the golden age of science is an understatement. We have more knowledge and technology than our predecessors could ever wish to have. On the other hand, previous clinicians and scientists would not envy the endemic chronic diseases that both developing and developed countries are facing. It will require a conscious change in both physician's and patients' mentality and thinking to counteract these disease trends. We need to stop looking at food as just calories and start looking at food as a flexible spending account to be used for the betterment of our health and quality of life.
